# Multi-Segmentation Parallel CNN Model for Estimating Assembly Torque Using Surface Electromyography Signals

**DOI:** 10.3390/s20154213

**Published:** 2020-07-29

**Authors:** Chengjun Chen, Kai Huang, Dongnian Li, Zhengxu Zhao, Jun Hong

**Affiliations:** 1 School of Mechanical and Automotive Engineering, Qingdao University of Technology, Qingdao 266520, China; dragonueo@163.com (K.H.); dongnianli@qut.edu.cn (D.L.); Zhaozhengxu@qut.edu.cn (Z.Z.); 2 Key Lab of Industrial Fluid Energy Conservation and Pollution Control, Ministry of Education, Qingdao University of Technology, Qingdao 266520, China; 3 School of Mechanical Engineering, Xi’an Jiaotong University, Xi’an 710049, China; jhong@mail.xjtu.edu.cn

**Keywords:** deep learning, assembly monitoring, surface electromyography signals, torque estimation, multi-segmentation parallel CNN model

## Abstract

The precise application of tightening torque is one of the important measures to ensure accurate bolt connection and improvement in product assembly quality. Currently, due to the limited assembly space and efficiency, a wrench without the function of torque measurement is still an extensively used assembly tool. Therefore, wrench torque monitoring is one of the urgent problems that needs to be solved. This study proposes a multi-segmentation parallel convolution neural network (MSP-CNN) model for estimating assembly torque using surface electromyography (sEMG) signals, which is a method of torque monitoring through classification methods. The MSP-CNN model contains two independent CNN models with different or offset torque granularities, and their outputs are fused to obtain a finer classification granularity, thus improving the accuracy of torque estimation. First, a bolt tightening test bench is established to collect sEMG signals and tightening torque signals generated when the operator tightens various bolts using a wrench. Second, the sEMG and torque signals are preprocessed to generate the sEMG signal graphs. The range of the torque transducer is divided into several equal subdivision ranges according to different or offset granularities, and each subdivision range is used as a torque label for each torque signal. Then, the training set, verification set, and test set are established for torque monitoring to train the MSP-CNN model. The effects of different signal preprocessing methods, torque subdivision granularities, and pooling methods on the recognition accuracy and torque monitoring accuracy of a single CNN network are compared experimentally. The results show that compared to maximum pooling, average pooling can improve the accuracy of CNN torque classification and recognition. Moreover, the MSP-CNN model can improve the accuracy of torque monitoring as well as solve the problems of non-convergence and slow convergence of independent CNN network models.

## 1. Introduction

Intelligence and mass customization have emerged as recent trends in manufacturing factories [1]. Although various types of robots and automation equipment have been replacing human workers in assembly operation, manual assembly is still indispensable for complicated or changeable assembly operation. In this case, the monitoring of the manual assembly process plays an important role in enhancing the production efficiency and product quality [2]. Bolted connection is one of the most common types of assembly connection. During the process of bolt assembly, if the tightening torque of the bolts does not meet the standards, the quality or performance of the products may be affected, even leading to product failure.

Different types of torque wrenches are frequently used in the bolt assembly to enable the operator to adjust the tightening torque according to the type of assembly process. In mass customization, the tightening torque of various products (even different bolts in the same product) varies, which requires the operator to frequently alter the tightening torque. This adversely affects the effectiveness of the assembly. In addition, using a common torque wrench, the torque cannot be monitored in real time during the tightening process of the bolt. Although some torque wrenches facilitate real-time torque measurement, they are large and heavy and not easy to use; hence, they are usually used in a laboratory only. Many methods have been developed to measure bolt stress, such as those using ultrasonic waves, resistance strain gages, piezoelectric impedance, and magneto-resistors. However, these methods are usually used to measure the stress of bolts after finishing the assembly or during the running of the product. Shirwalker et al. proposed a wrench perception method in intermediary telepresence for remote manipulation, wherein the force diagram of the wrench is obtained to present the minute details of the interaction of the wrench at the robot end-effector with the environment [3].

This paper proposes a new method using surface electromyography (sEMG) signals for measuring and monitoring the torque during the tightening of bolts. In the proposed method, the operator wears a sEMG signal acquisition device called a Myo armband on his/her arm during the assembly process. The Myo armband is wireless and light, which makes the proposed method suitable for use in factories. A two-dimensional (2D) convolution neural network (CNN) model is used to extract torque values from sEMG signals. The proposed method can monitor the assembly process of bolt tightening in real time and is suitable for monitoring the product assembly quality in a mass customization setting.

The following are the contributions of this study:

(1) A multi-segmentation parallel convolution neural network (MSP-CNN) model for estimating assembly torque using sEMG signals is proposed in this paper. This model consists of two separate CNN models with a different number of segmentations; the output of each CNN model is fused to obtain accurate torque estimation. Compared with the torque accuracy obtained by the separate CNN models, the obtained by the proposed MSP-CNN model is significantly improved.

(2) The proposed method for wrench torque monitoring uses a wearable myoelectric measurement device called the Myo armband to measure the sEMG signals generated by the operator performing bolt tightening. The Myo armband is compact and communicates with a computer wirelessly (via Bluetooth). Therefore, the proposed system can monitor torque of the wrench in the assembly operation of products without increasing the burden on operators, thus showing certain practical value in assembly monitoring and automation areas.

(3) A bolt-tightening test bench is designed. In the test, the sEMG signals are collected to identify the assembly torque signals and the assembly tightening dataset is established through signal preprocessing.

This paper is organized as follows. Section 2 summarizes the state-of-the-art techniques of sEMG and their applications. Section 3 describes the flowchart of the torque monitoring process and the specific structure of the MSP-CNN model. In addition, the methods for acquiring and preprocessing sEMG and torque signals are elaborated. In Section 4, experimental tests that demonstrate the effectiveness and efficiency of our method are presented. Section 5 presents our conclusions and future work.

## 2. Related Work

EMG signals can reflect the characteristics of human muscle activation and muscle strength [4,5]. sEMG is a non-invasive technique for measuring electrical signals generated by the contraction of skeletal muscles. The obtained signals are one-dimensional (1D) temporal signals that reflect neuromuscular system activity [6,7]. sEMG signals are widely applied in rehabilitation medicine, human–computer interaction, action recognition, and other fields. For example, sEMG signals generated by the user’s muscles are used to estimate muscle force and recognize the motion type of the joint, which is then used to control a power-assisted exoskeleton arm [8]. The state-of-the-art action recognition and force estimation research based on sEMG signals is summarized as follows.

In the field of action recognition, Khezri et al. [9] proposed a method for recognizing hand movements using sEMG. To increase the accuracy of the system and to obtain more signal information from each movement, time features, time–frequency features, and their combination were used. In addition, two classifiers, namely the artificial neural network (ANN) and fuzzy inference system, were used to recognize eight hand movements. Compared to hand movement classification, finger movement classification is more difficult because sEMG signals of finger movements are weaker. Al-Timemy et al. [10] proposed a classification method of finger movements for the dexterous control of prosthetic hands. In this method, multichannel sEMGs were used to classify 15 classes of finger movements. Through time–domain autoregression feature extraction, orthogonal fuzzy neighborhood discriminant analysis for feature reduction and linear discriminant analysis for classification were conducted and high classification accuracies with minimum latency of 200 ms were achieved. Gaudet et al. [11] classified upper limb phantom movements in amputees using EMG and kinematic features. An ANN model was employed to classify eight main upper limb phantom movements and the case of no movement. Their study revealed that adding a kinematic feature can enhance the average accuracy by 4.8%. All abovementioned studies focus on motion recognition using sEMG signals and usually adopt intelligent classification methods such as neural networks as a classifier. However, they can only determine the type of motion based on sEMG signals and cannot achieve motion amplitude identification. Therefore, the research on extracting quantitative muscle strength from sEMG signals gradually developed.

The sEMG signal is highly correlated to muscle strength, and estimation of the force generated by an activated muscle has many practical applications in the biomechanical and clinical fields [12]. Staudenmamnn et al. [13] reviewed some methods for estimating muscle force using sEMG and pointed out that high-pass filtering of EMG signals can improve the precision of EMG amplitude estimates and that multichannel monopolar EMG can be used to improve estimation accuracy. These methods assist in sEMG signal processing and muscle strength estimation. Li et al. [14] applied multifractal detrended fluctuation analysis on the nonlinear characteristics of correlation between operation force and sEMG signals. Cross-correlation functions between the force signal and four typical sEMG time–frequency domain index sequences were established. They demonstrated that the force–sEMG cross-correlation sequences have strong statistical similarity.

Methods for extracting force from EMG signals can be classified into two categories: parametric model-based algorithms and nonparametric algorithms [15]. The parametric model-based algorithms [16,17] require accurate parameters of muscle or an accurate muscle-based model, so their applications in some important fields are restricted [15]. Therefore, nonparametric algorithms have gained increasing attention as they do not require prior knowledge of the parameters of arm muscle models. For example, Cao et al. [18] applied an extreme learning machine (ELM) to predict handgrip force from sEMG signals of forearm muscles. Their experimental results demonstrated relatively good accuracy and time requirement of the ELM. To consider the effect of arm posture on grasping force extraction from sEMG signals, Kim et al. [19] proposed an algorithm to predict the grasping force from sEMG signals and the grasping posture. Tensor algebra was used to train a multi-factor model relevant to sEMG signals corresponding to various grasping forces and postures of the wrist and forearm in multiple dimensions. The multi-factor model was then decomposed into four independent factor spaces, namely the grasping force, sEMG signals, wrist posture, and forearm posture.

Different types of neural networks have been widely employed to measure the muscle force using EMG signals. Luo et al. [15] used a three-domain fuzzy wavelet neural network algorithm without the requirement of prior knowledge of the biomechanical model to accurately estimate the human arm force from EMG signals. ANN regression models were used to estimate hand force from sEMG signals. The test results showed that ANNs with deeper layers of up to four hidden layers show fewer errors in intrasession and intrasubject evaluations. Deep learning methods are presently used to estimate force from EMG signals. Xu et al. [20] applied a CNN, a long short-term memory (LSTM) network, and their combination (C-LSTM) to estimate the muscle force generated in static isometric elbow flexion. The accuracy, real-time performance, and subject-independent performance were tested and compared. The test results found the CNN model to perform the worst with the largest root mean square error (RMSE). Dao [21] proposed a recurrent deep neural network incorporating dynamic temporal relationships to estimate skeletal muscle forces from kinematics data during a gait cycle. In addition, a transfer learning strategy was used to improve the accuracy of muscle force estimation.

Most of the abovementioned nonparametric algorithms are regression-based algorithms. To the best of our knowledge, few classification-based neural networks are used to estimate force from EMG signals, mainly because these networks result in large errors and low accuracy of force estimation. Leone et al. [22] introduced a hierarchical classification approach to assess the desired force exerted for grasping tasks. To ease the difficulty in estimating an accurate force value, they defined three force levels (low, medium, and high levels) according to the peak force. Their experimental results revealed that the use of the nonlinear classification algorithm is as much suitable as the benchmark linear discriminant analysis classifier for implementing an EMG pattern recognition system.

Inspired by the studies on the application of CNN models for multiscale image feature learning [23] and image super-resolution [24], in this study, a classification-based method is proposed to estimate assembly torque from sEMG signals. Recently, a parallel CNN method was used in hand gesture recognition to obtain multi-resolution features of the hand [25] and in high-spatial-resolution image land-cover classification [26]. Hence, in order to further improve the accuracy of torque estimation, a parallel CNN model including two separate CNN models with multiple segmentations (hereafter MSP-CNN) is proposed. The output of each CNN model is fused to obtain a more accurate estimation torque.

## 3. Methods

### 3.1. Framework

**Figure 1:**
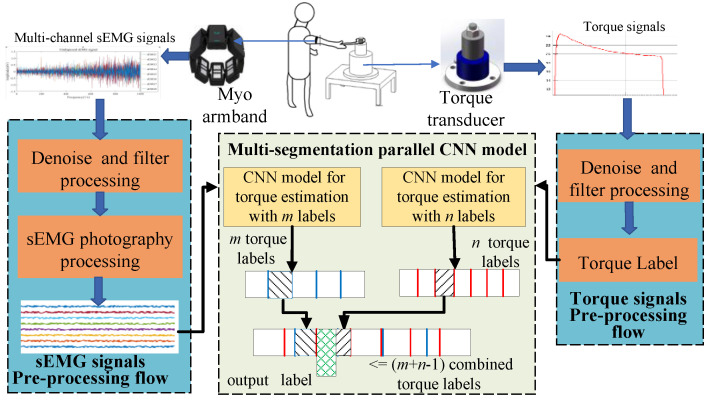
Research framework and the architecture of the multi-segmentation parallel convolution neural network (MSP-CNN) model.

The research process and the architecture of the proposed MSP-CNN model are shown in Figure 1. First, a Myo armband is worn by the assembly operator for collecting the sEMG signals of his or her arm when tightening a bolt using a wrench. In addition, a torque transducer provides signals of the tightening torque of the wrench. Further, the sEMG and torque signals are separately processed to establish sample data sets for neural network training and real-time torque monitoring. Then, the data sets are adopted to train the MSP-CNN model and determine torque values. The specific steps involved in the entire process are as follows.

(1) A bolt tightening test bench is designed, which is mainly composed of a torque transducer, a wearable sEMG signal acquisition device, called the Myo armband [27], a torque monitoring system based on the EtherCAT bus, and a computer. Using the test bench, the sEMG and torque signals generated by the operator during assembly tightening are collected and recorded.

(2) According to the data characteristics, the sEMG and torque signals are preprocessed separately. The sEMG signals are converted to EMG data according to the time sequence, and the torque signals are converted to torque labels corresponding to the EMG data; thus, the assembly tightening datasets are established.

(3) The assembly tightening datasets are randomly divided into a training set, a verification set, and a test set. To establish a CNN model, the training and verification sets are applied to train the CNN, and the CNN model structure and parameters are optimized. The test set is used to verify the optimized CNN model, and the training accuracy and generalization of the network are determined.

(4) Next, the structure of the MSP-CNN model is designed, the estimation effects of the CNN are analyzed, the error values between the estimated torque results and the actual collected torque results are compared under different or offset torque classification granularities, and the MSP-CNN model is adopted to improve the torque monitoring accuracy.

### 3.2. MSP-CNN Model

The structure of the proposed MSP-CNN model is shown in Figure 1. The parallel CNN model contains two independent CNN models, whose inputs are EMG graphs and the outputs are labels for torque classification. Two independent CNN models employ the same EMG in the sample sets and torque labels with different granularities or offset granularities for training and optimization. The output torque labels of the two independent CNN models are then combined to obtain a finer classification granularity and improve the accuracy of torque estimation.

**Figure 2:**
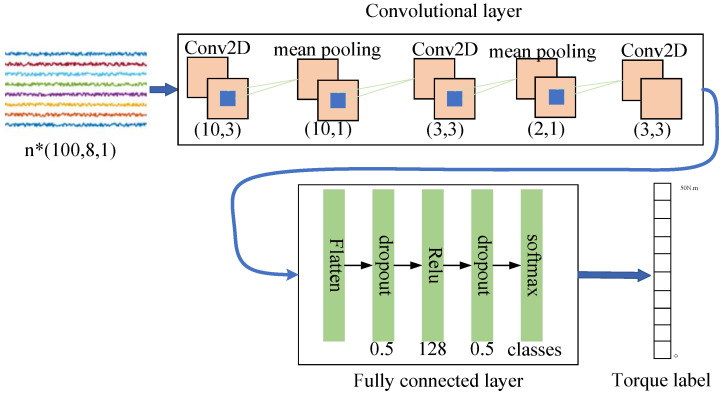
Framework of independent CNN.

The CNN network Figure 2 has certain advantages in handling 2D data classification. The input of the model is a 2D sEMG signal graph. After being processed by three convolutions and two pooling layers, the 2D data are reduced to 1D data by a flattening layer. The 1D data are then computed by operations including dropout and ReLU, and finally the junction torque estimation labels are output through the softmax layer. The specific steps involved are described as follows.

(1) Input layer: The input is a preprocessed EMG of size (100, 8, 1).

(2) Convolution layer: As shown in Figure 2, the convolution layer includes three convolution computations and two pooling computations. The purpose of convolution computations is to extract the features of EMG and add bias to it, and then conduct activation through the activation function [28]. The number of filters in the first convolution computation is 32, the size of the convolution kernel is (10, 3), the step size of the convolution computation is (1, 1), and the activation function uses ReLU [29]. The number of filters for the second convolution computation is 64, the size of the convolution kernel is (3, 3), the step size of the convolution computation is (1, 1), and the activation function is ReLU. Furthermore, the number of filters for the third convolution computation is 128, the size of the convolution kernel is (3, 3), the step size of the convolution computation is (1, 1), the activation function uses ReLU, and the filling layer uses VALID [30]. The pooling computation, also called down-sampling computation, aims to reduce the parameters while preserving the main features, reduce the phenomenon of overfitting, and improve the generalization ability of the model. Based on performance comparison, average pooling [31] is adopted because sEMG signals are a signal sequence, and it is assumed that max pooling may affect the continuous characteristics of signals. The pooling size of the first pooling computation is (10, 1) and that for the second pooling computation is (2, 1).

(3) Fully connected layer: The fully connected layer includes flatten computations, two dropout computations, and fully connected neural network computations. Flattening computations reduce the 2D data after Conv2D into 1D date that can be an input for the next computations. Dropout calculation [32] solves the phenomenon of easy overfitting caused by fewer training samples and more model parameters. The fully connected neural network classifies and calculates 1D data according to the set labels, where the number of the filters is 128, and the activation function uses ReLU.

(4) Softmax layer: The Softmax layer is used as the output layer to output the torque classification results and map the output results to (0, 1), representing the probability value of the classification results.

### 3.3. Establishment of the Assembly Tightening Dataset and Data Preprocessing

#### 3.3.1. Establishment of the Assembly Tightening Datasets

**Figure 3:**
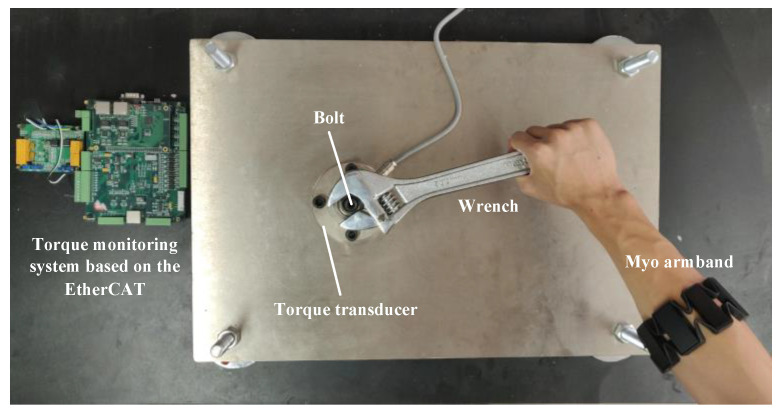
Bolt tightening test bench.

**Figure 4:**
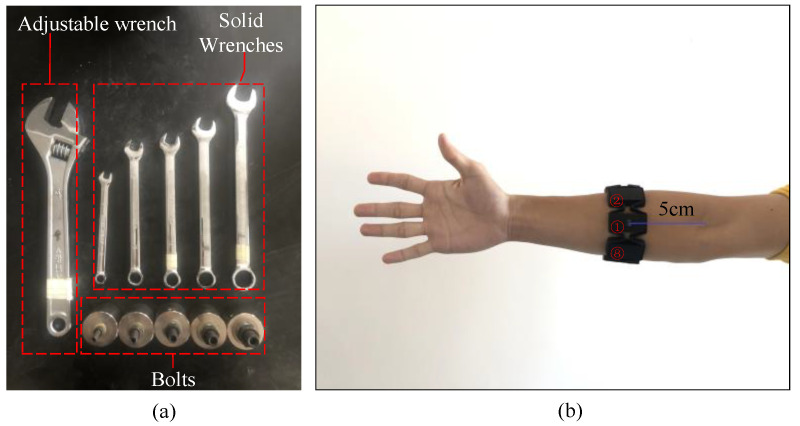
Schematic of bolt assembly tools and Myo armband wearing. (**a**) wrench and bolts used in this test; (**b**) the Myo armband worn on the arm of an operator.

As public datasets for the monitoring of torque based on sEMG signals are not available, a bolt tightening test bench is designed to establish torque monitoring datasets based on sEMG signals for CNN training. The dataset includes a total of 4000 sEMG signals and its corresponding torque labels. The structure of the bolt tightening test bench is shown in Figure 3. The operator simulates the bolt tightening process performed in the factory. Moreover, the sEMG signals generated by the operator and the bolt torque signals during bolt tightening are recorded by the Myo armband and the torque transducer, respectively, along with a control system based on the EtherCAT bus. During the experiment, to collect sEMG signals, the operator wears the Myo armband [27], which is a myoelectric hand-gesture recognition armlet consisting of eight interconnected electrodes. The Myo armband can be used for eight-channel electrical signal acquisition at a frequency of 200 Hz in different directions. In order to match the eight-channel sEMG signals, the Myo armband should be worn correctly before the experiment; this can avoid inconsistent data caused by factors such as the deviation of the wearing position of the Myo armband, which can affect the training accuracy and even cause distortion. As shown in Figure 4, in this experiment, the electrode with the Myo logo is marked as electrode 1. Electrode 1 is located on the inside of the arm and aligned with the middle finger at distance of 5 cm from the elbow. The semi-circle of the logo points outward. The other electrodes are marked in the clockwise order.

The selection criteria for operators who participated in the experiment were as follows: First, operators should have no history of arm-related diseases. Second, they should be familiar with bolt assembly operations. Third, the sEMG signal feedback received from the Myo armband worn by operators should be clear. A total of 10 operators (males, aged 23 ± 3 years) participated in the experiment to establish the dataset. The body mass indexes of the operators, as per the international standard, were 21.6, 21.7, 23.0, 27.7, 23.1, 22.3, 19.1, 19.6, 22.5, and 23.5. Note that one of the operators was overweight and the others were normal. The author of this paper promised to keep the name information of the operators participated confidential, and the experimental data should not be used for nonacademic purposes.

Each operator used different assembly tools to tighten different types of bolts (i.e., M6, M8, M10, M12, and M14) on the test bench, as shown in Figure 4. The tightening tools used were an adjustable wrench and five solid wrenches of various sizes. Each operator used both types of wrenches to tighten the five bolts for four experiments; the time required to tighten the bolts each time was 5 s. During the experiment, the sEMG and torque signals were collected simultaneously. After data preprocessing, the sEMG signals were processed into 4000 EMG graphs and the torque signals were processed to produce torque labels corresponding to the EMG graphs. These were marked as the required dataset for this experiment. The dataset can be downloaded from https://github.com/QDLGARIM/torque_semg_dataset.

#### 3.3.2. Preprocessing of sEMG Signals

**Figure 5:**
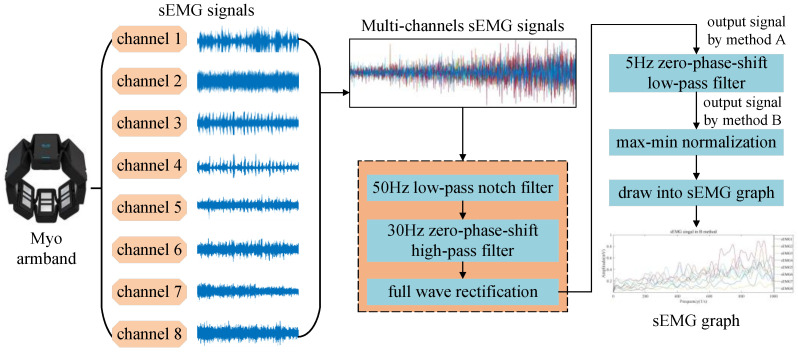
Preprocessing flow of surface electromyography (sEMG) signals.

The preprocessing of the collected sEMG and torque signals will improve the accuracy of the CNN calculation process [33]. The processing flow of sEMG signals is shown in Figure 5, and the specific steps are as follows.

(1) A 50-Hz low-pass notch filter [34] is used to process the sEMG signals to eliminate the interference caused by the local power frequency in the sEMG signals.

(2) A 30-Hz zero-phase-shift high-pass filter [35] is used to eliminate the noise generated by manual operation in the sEMG signals processed in Step 1.

(3) The negative values of the signals are inverted for full wave rectification. After this operation, the signal output by the system is recorded as the output signal by method A (hereafter method A).

(4) Output signal A is filtered by a 5-Hz zero-phase-shift low-pass filter to simulate the low-pass filter characteristics of muscles [36]. After this operation, the signal output by the system is recorded as the output signal by method B (hereafter method B).

(5) Max–min normalization of the eight-channel sEMG signals is performed as given by Equation (1) [37], and the sEMG signal amplitudes are mapped to a range [0,1] to convert the sEMG signals into scalar data and eliminate the effects of vectors.

(1) $$A_{i}^{\prime }=\frac{A_{i}-A_{min}}{A_{max}-A_{min}}$$

Here, *A_max_* and *A_min_* are the maximum and minimum values of the sEMG signals in eight channels, respectively. *A_i_* is the *i*-th sEMG value, and *A_i_*′ is the max-min normalized value of the *i*-th sEMG signal.

(6) According to every 100 values (because the sampling frequency of the sEMG signals is 200 Hz), which is a group of sEMG signals in 0.5 s, the normalized sEMG signals are drawn into one EGM graph. Thus, a total of 4000 EMG graphs are obtained, with each having a size of (100, 8), where 8 represents the eight channels of sEMG signals.

#### 3.3.3. Preprocessing of Torque Signals

The collected torque signals are processed to generate labels as follows.

(1) The torque signals are normalized according to Equation (2) to obtain *T_i_*′

(2) $$T_{i}^{\prime }=l_{max}T_{i}/l_{i}$$

where *l_max_* is the length of the longest wrench, *l_i_* is the length of the *i*-*type* wrench, *T_i_* is the torque measured under the *i*-*type* wrench, and *T_i_*′ is the standardized torque under the *i*-*type* wrench.

(2) A 50-Hz low-pass notch filter is applied to the torque signals to eliminate the effects of local power frequency on the signals.

(3) A 30-Hz zero-phase-shift high-pass filter is applied to eliminate the motion traces of the standardized torque signals.

(4) To ensure the correspondence between the torque eigenvalues and the EMGs in time sequence, the EMG length is set as 0.5 s and the average value of the torque signals every 0.5 s is calculated as the average torque.

(5) The torque subdivision granularity is set. The range of the torque transducer used in this study is 0–50 N·m. The torque values within the range are divided into the following 10 torque ranges (considered as 10 granularity equalizations): [0, 5), [5, 10) [10, 15), [15, 20), [20, 25), [25, 30), [30, 35), [35, 40), [40, 45), and [45, 50) (in N·m). According to the above method, the torque range can be equally divided into 5, 15, and 20 granularities.

(6) A one-hot coding method is applied to encode the torque after equipartition. This code is the torque label corresponding to the sEMG during CNN training.

**Figure 6:**
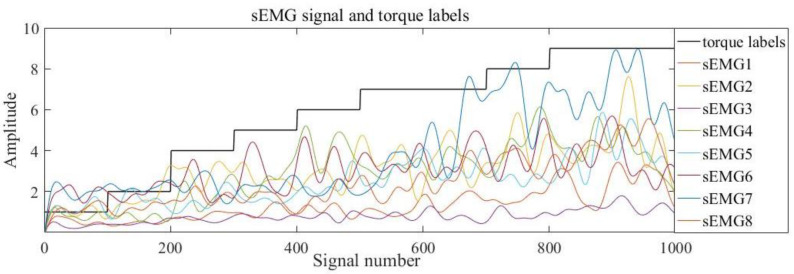
sEMG signals and torque labels.

Labels 1–20 are used to identify the torque range under different classification granularities. Taking the classification granularity 10 as an example, label 1 represents the torque range [0, 5). Figure 6 shows the sEMG output signals and the corresponding torque labels after preprocessing. The Figure indicates that a certain relationship exists between the torque labels and the amplitude of the sEMG signals.

## 4. Experiment and Result Analysis

The CPU used in this experiment is an Intel® Core™ i5-8300H CPU @ 2.30 GHz × 4, and the GPU is an NVIDIA GeForce GTX 1050Ti. Furthermore, the operating system is Ubuntu 18.04 (64 bit), with an NVIDIA Quadro M4000 graphics card. First, the effects of the sEMG signal preprocessing methods and the pooling methods of the independent 2D CNN model was analyzed using a small sample dataset (800 electromyographs). Second, the optimal method was selected for torque estimation of different classification granularities using the total dataset (4000 electromyographs), and the corresponding estimation error was analyzed. Third, the MSP-CNN model was applied on the datasets and the obtained results were compared with those of the 2D CNN model.

### 4.1. Analysis of sEMG Signal Preprocessing Methods

**Figure 7:**
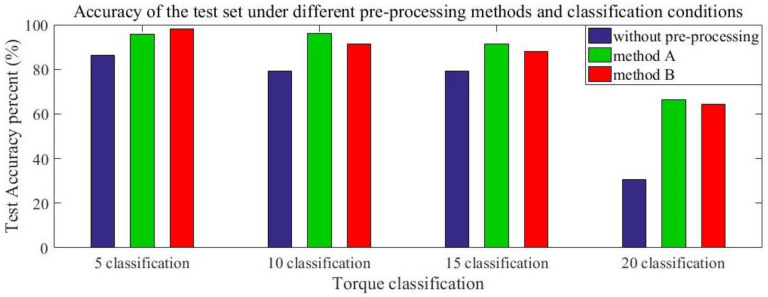
Accuracy of the test set under different preprocessing methods and classification conditions.

In this study, the original sEMG signals, the sEMG signals processed by method A, and the sEMG signals processed by method B Figure 5 were used to train the CNN model with the same batch processing size and training times. Moreover, the accuracies of both the training set and the test set under different torque subdivision granularities were calculated. The independent CNN network adopted the structure described in Figure 2. Table 1 and Figure 7 show the accuracies of the training set and test set of different preprocessing methods under the conditions of 5, 10, 15, and 20 torque classification granularities.

**Figure 8:**
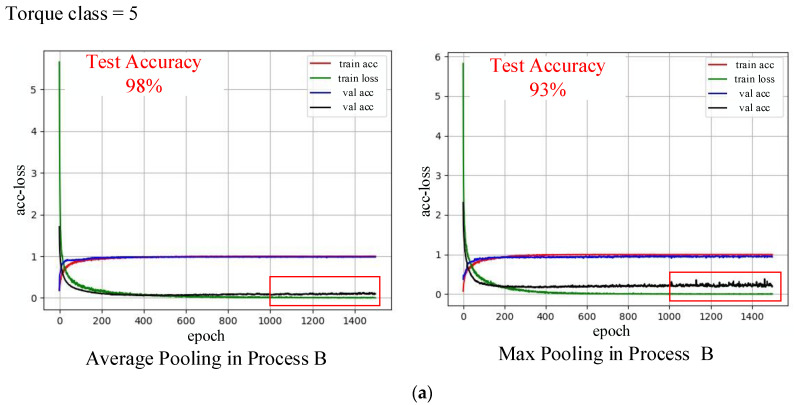

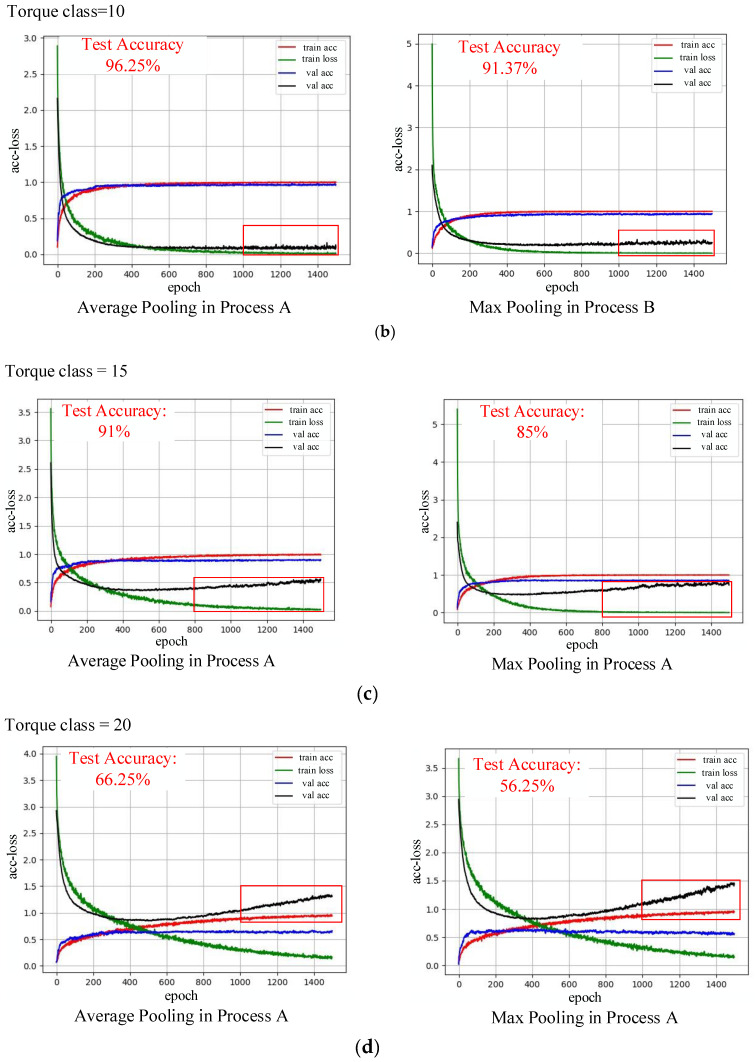
Effects of pooling methods on network training under various classification granularities: (**a**) 5, (**b**) 10, (**c**) 15, (**d**) 20.

Table 1 and Figure 7 indicate through comparison that preprocessing (methods A and B) on the sEMG signals can significantly improve the prediction accuracies, increase the average accuracy of the test set by approximately 15%, and improve the accuracy of torque recognition. Although a 5-Hz zero-phase-shift low-pass filter processing has been proven to simulate muscle characteristics [36], compared with the torque recognition accuracies of methods A and B, the 5-Hz zero-phase-shift low-pass filter processing had no significant effect on accuracy improvement. This is because in this experiment, the operator applied an intermediary tool (wrench), which indirectly increased muscle strength and offset the effect of power frequency interference. The finer the torque classification granularity, the lower the network recognition accuracy can be. When the torque classification granularity was very fine (such as 20), the independent CNN network had the worst training effect and the training process did not converge Figure 8, with an estimated accuracy of only 66.25%. This was because the Myo armband used in this study had a larger measurement error than the professional sEMG testing equipment used in other studies [38]. Therefore, too fine classification granularity would increase the training difficulty of the independent CNN model.

### 4.2. Analysis of CNN Model Pooling Methods

In this experiment, 800 electromyographs were used to train the CNN model. The effects of the average pooling and maximum pooling methods on the estimation accuracy of the CNN model were compared. As given by Equation (3) [39], the average pooling method calculates the average of all elements in the pooling size, which is used as the eigenvalue to represent the pooling size.

(3) $$M_{j}=\tanh(\beta \sum_{N\times N}M_{i}^{n\times n}+b)$$

Here, *M* is the input of the pooling layer, $M_{i}^{n\times n}$ is a sub-matrix of *M*, *b* is the bias, and βis a trainable scalar.

As given by Equation (4) [39], the maximum pooling method selects the maximum value in the pooling size, which is regarded as the eigenvalue representing the pooling size.

(4) $$M_{j}=\underset{N\times N}{\max}(M_{i}^{n\times n}u(n,n))$$

The average pooling can increase the connection between pooling sizes, extract eigenvalues more smoothly, and improve the connection between pooling sizes, while the maximum pooling is more advantageous in processing image information.

As Figure 8 shows, according to the optimal sEMG signal preprocessing method determined with the aforementioned different torque classifications, method A was used for torque classification 5 and method B was used for torque classifications 10, 15, and 20. Under the conditions of the same training times and the same batch processing size, the effects of the average pooling and maximum pooling were compared. It was found that the average pooling method is superior to the maximum pooling method in terms of estimation accuracy and convergence speed. To reduce the effects of the epoch on training, an early stop mechanism is added in the 2D CNN. Therefore, an overfitting phenomenon was observed for classifications 15 and 20 with increasing training times.

### 4.3. Analysis of CNN Model Torque Estimation Errors

The torque classification granularity was refined and all datasets (4000 electromyographs) were applied for testing. The torque estimation errors obtained using different classification granularities were compared.

**Figure 9:**
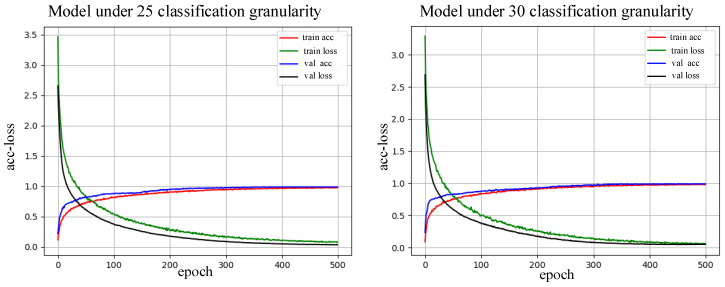

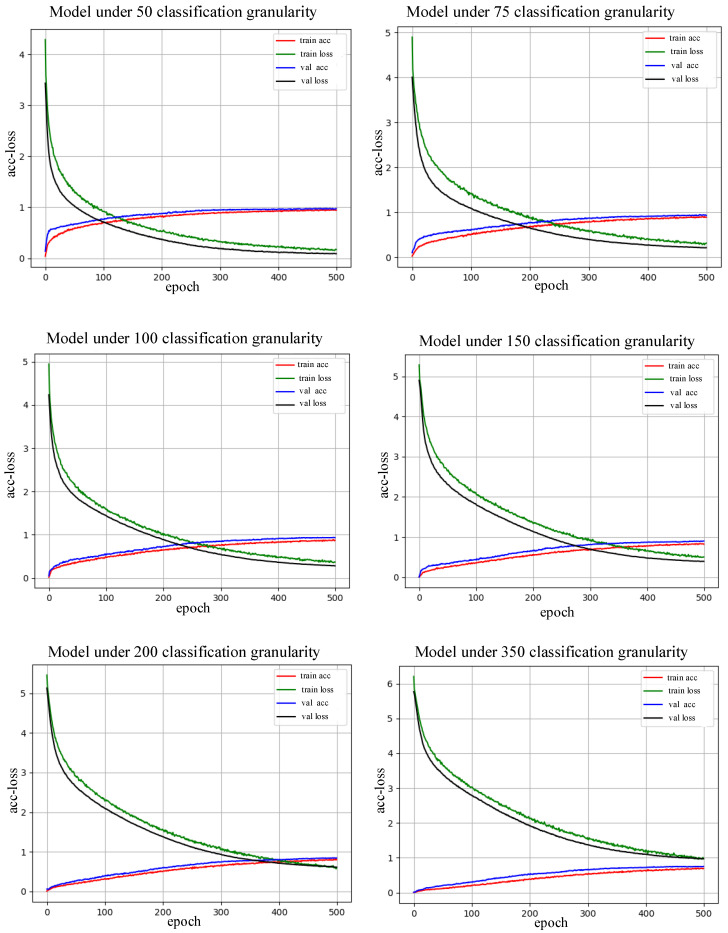

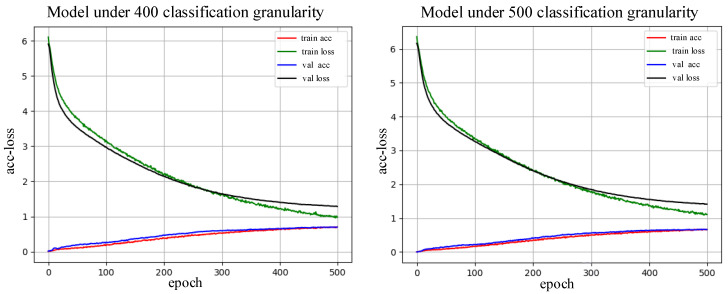
Effect of curve fitting under different classification granularities.

Figure 9 shows that the curve fitting is better and the accuracy is higher when the 2D CNN network is trained with sufficient datasets. Further comparison of the training curves of the 2D CNN model using torque classification granularities 25, 30, 50, 75, 100, 150, 200, 350, 400, and 500 indicates that with greater classification granularity, the model fitting is slower and the required training time is longer.

The torque estimation error is one of the main indicators of the evaluation of the CNN model. Hence, error analysis on the estimated torque was performed. The torque estimation accuracy can be improved and the estimation error can be reduced by analyzing the torque classification accuracy, torque estimation error, convergence speed, and the selection of the optimal classification granularity.

**Figure 10:**
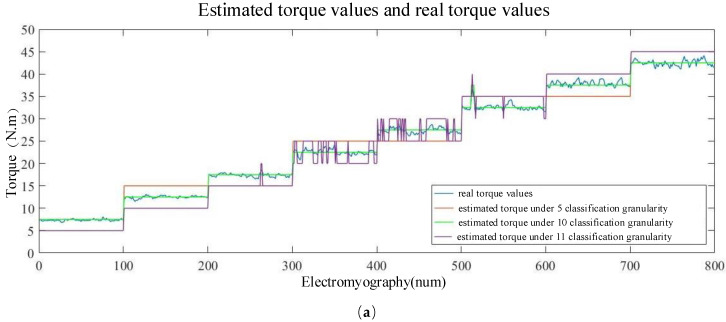

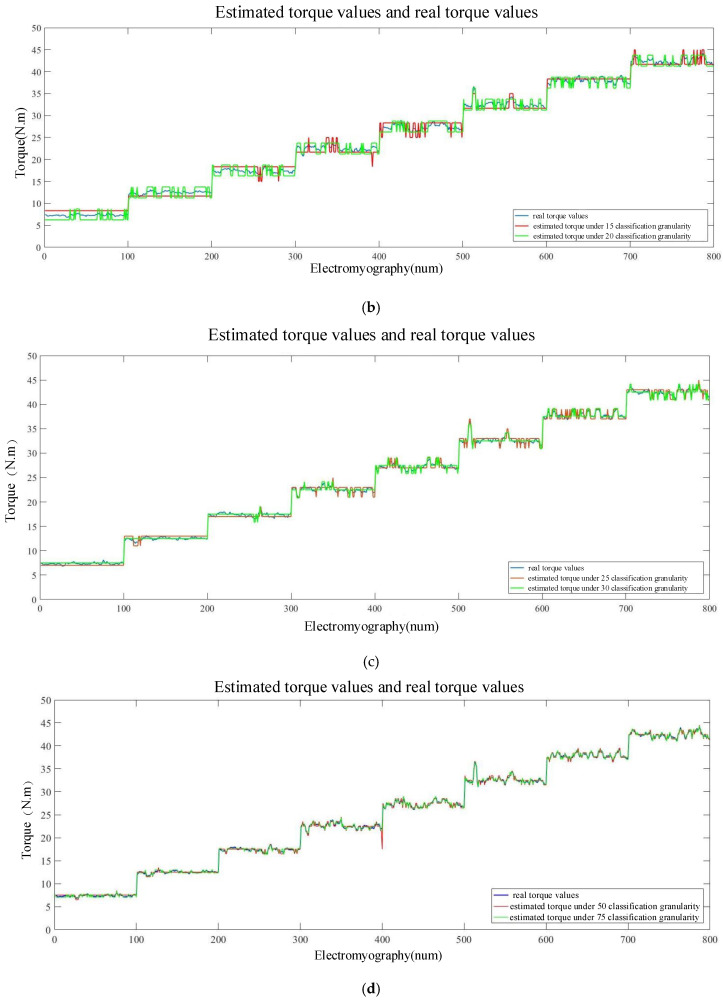

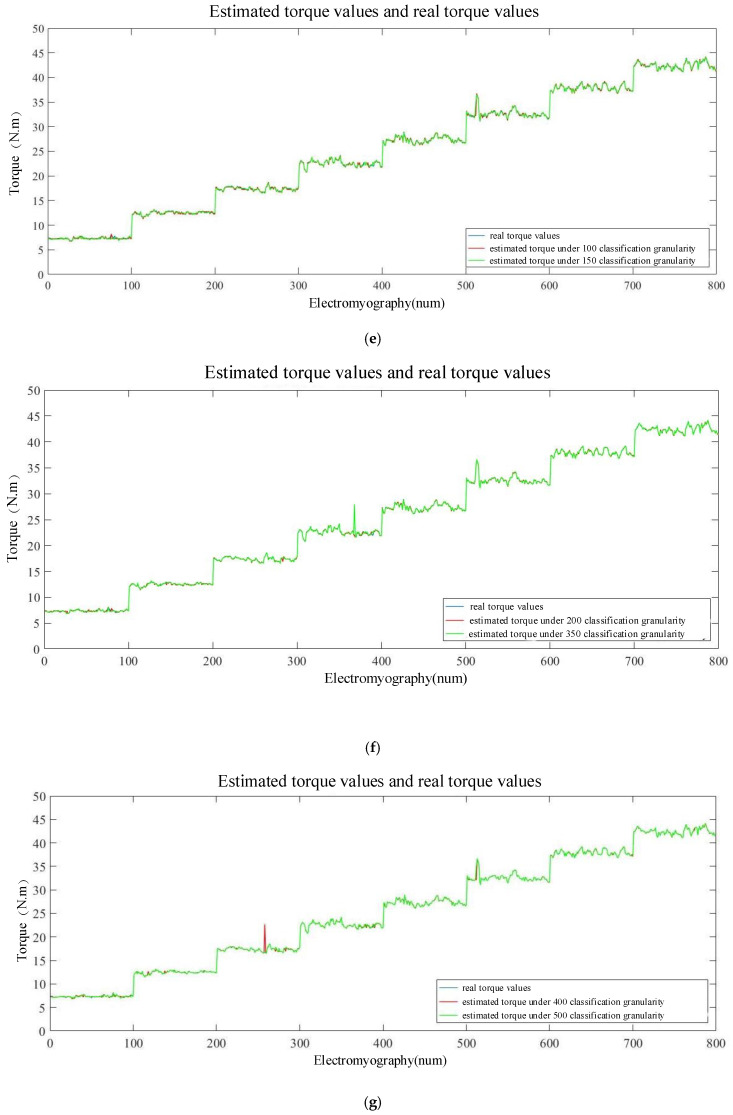
Estimated torque values and real torque values under different granularities: (**a**) 5, 10, and 11; (**b**) 15 and 20; (**c**) 25 and 30; (**d**) 50 and 75; (**e**) 100 and 150; (**f**) 200 and 350; (**g**) 400 and 500.

First, 800 sEMG graphs and their corresponding torque labels were randomly selected from the test set for error analysis. For different classification granularities, the optimal preprocessing method and the optimal CNN structure were selected to perform estimation analysis. Figure 10 shows the estimated torque values and the real torque values using different torque classification granularities. It can be observed that the torque estimation error can be reduced by refining the torque classification granularity. However, the maximum torque estimation error would increase under the same training epoch due to the over-fine classification granularity, thus affecting the judgment in the assembly bolt process. 

To calculate the torque estimation error, this paper divided the torques into different granularities. First, the average of the torque values of all training set samples in each label was calculated and recorded as the eigenvalue *T^k^* of the corresponding label.

(5) $$T_{}^{k}=\frac{\sum_{j=1}^{N_{k}}T_{j}}{N_{k}}$$

where *N_k_* is the total number of samples with torque label *k* in the training set.

Subsequently, by calculating the difference between the actual torque value and the corresponding eigenvalue of the estimated torque label, the estimation error was obtained as follows.

(6) $$\Delta T=\frac{\sum_{i=1}^{N}\left|T_{i}-T_{}^{k}\right|}{N}$$

where Δ*T* is the error value, *T_i_* is the real torque value corresponding to the *i*-th EMG in the test set, *N* is the total number of samples in test set for error analysis, and *T^k^* is the eigenvalue corresponding to estimated torque label k of the *i*-th EMG in the test set.

Table 2 lists the torque errors calculated according to Equation (6) using different torque classification granularities. The results indicate that the error for classification granularity 10 was the smallest, mainly because the torque value was artificially controlled to fluctuate around an integer multiple of five when the sample set was established to collect torque signals Figure 10a. Therefore, classification granularity 11 was used instead of 10. The coarser the classification granularity, the greater was the torque estimation error. However, at the same time, the accuracy of the model was higher and fitting occurred faster. The torque estimation effect was better for fine classification granularity, but the accuracy of the model decreased and the maximum error worsened. Thus, the proposed MSP-CNN model not only ensured the rapid convergence of the model, but also improved the torque estimation accuracy.

### 4.4. Analysis of MSP-CNN Model Results

The MSP-CNN model first performed independent CNN training and testing on the two torque classification granularities and then combined the outputs of the independent CNN models under the two classification granularities to form a finer classification granularity. If the same sEMG graph is input, and the eigenvalues corresponding to the maximum probability torque labels output by two independent CNN models are *T_m_* and *T_n_*, respectively, then (*T_m_* + *T_n_*)/2 is used as the torque estimation of the MSP-CNN model.

As Table 3 shows, in the MSP-CNN model, two different classification granularities are combined into a new torque classification granularity, which reduces the torque estimation errors.

**Figure 11:**
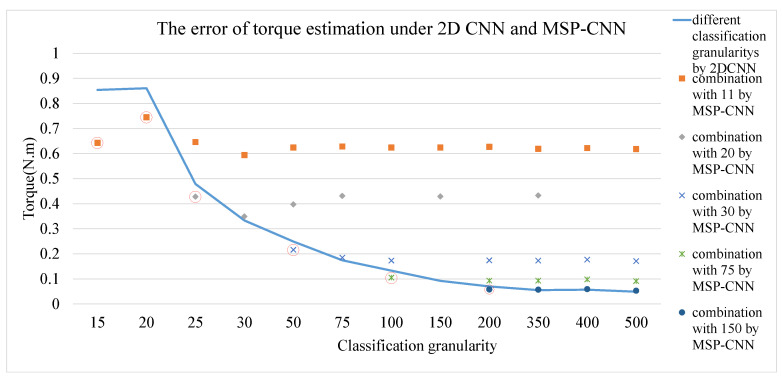
Torque estimation error under two-dimensional (2D CNN) and MSP-CNN.

Figure 11 shows that the torque estimation error of the MSP-CNN model is lower than that of the 2D CNN model when two torque classification granularities of the single CNN are similar. However, the effect of MSP-CNN will gradually weaken with a large difference between the two torque classification granularities.

When the difference between two different classification granularities is large, the optimization effect of the MSP-CNN model is not obvious. Therefore, the MSP-CNN model was improved by using the same classification granularity with an offset of half of the torque label range. For example, in MSP-CNN model using five classification granularity, the torque range of a torque label is 10 N·m (this is because that the range of the torque transducer used in this study is 0–50 N·m). The first torque labels of two independent 2D CNN models are from 0–10 N·m and 5–15 N·m respectively. If the same sEMG graph is input and the eigenvalues corresponding to the maximum probability torque labels output by two independent 2D CNN models are *T_m_* and *T_m_*′, respectively, then (*T_m_* + *T_m_*′)/2 is used as the torque estimation of the new MSP-CNN model. As Table 4 shows, compared with the 2D CNN, the MSP-CNN can significantly reduce the torque estimation error. For a classification granularity of 25, the torque estimation error obtained by the MSP-CNN was 0.243 N·m, which is lower than the error obtained by the 2D-CNN.

**Figure 12:**
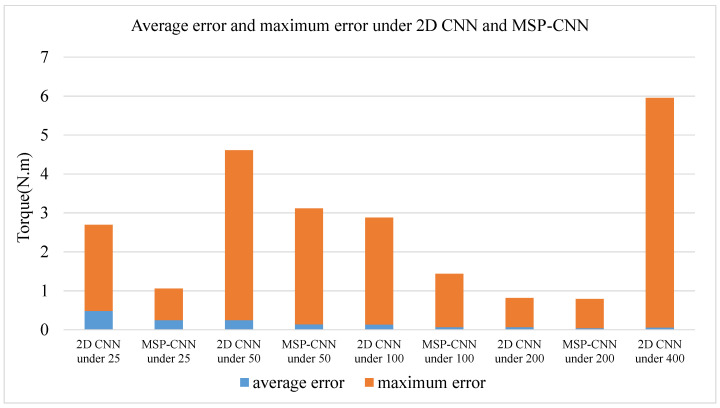
Average error and maximum error of MSP-CNN and 2D CNN.

Figure 12 shows the average error and the maximum error of the 2D CNN and MSP-CNN. Compared with the 2D CNN, both errors of the MSP-CNN are lower.

### 4.5. Discussion

To monitor the assembly process of bolt tightening in real time, this study proposed a new method using sEMG signals for measuring and monitoring the torque during the tightening of bolts. In the proposed method, the operator wears a sEMG signal acquisition device called a Myo armband on his/her arm during the assembly process. The preprocessing method of sEMG signals and the pooling method of the CNN model were optimized by training the 2D CNN model using a small dataset. An MSP-CNN network was proposed by combining two close or the same torque classification granularities. The test results showed that the MSP-CNN model can reduce the average error and the maximum error (Table 4). Unlike the existing studies, this study mainly applied sEMG signals to mechanical assembly processing to achieve assembly torque estimation and proposed the MSP-CNN model that can reduce torque estimation errors. The proposed methods are suitable for monitoring the product assembly quality in a mass customization setting.

The main limitation of this study is that the sEMG signal responses of different persons may have slight differences. These differences can influence the estimation error of assembly torque. 

## 5. Conclusions and Future Work

In this study, sEMG signals were applied to monitor torque in the bolt tightening process. A bolt tightening test bench was then designed, based on which assembly tightening datasets were established. A 2D CNN framework for estimating assembly torque based on sEMG signals was constructed. This study used the classification method to extract the torque value from sEMG signals; furthermore, the MSP-CNN model was proposed to reduce the torque estimation errors. The experimental results confirmed that the application of sEMG signals can achieve real-time torque monitoring during the assembly process through 2D CNNs. The average pooling method is superior to the maximum pooling method in terms of estimation accuracy and convergence speed. The proposed MSP-CNN model can ensure improvement in the accuracy of torque estimation with rapid convergence of the 2D CNN models. The CNN classification model is used to solve the regression problem. The results of this study confirmed a certain reference value for bolt tightening torque monitoring as well as for numerical prediction based on CNN classification. sEMG signals are directly related to human muscle motion; however, in an assembly task, there may be uncertainties such as different assembly tools and actions. Subsequent studies will evaluate the influence of sEMG signal responses of different persons on estimation error of assembly torque and will incorporate other signals (such as inertial measurement unit movement signals and videos) to achieve greater accuracy of assembly torque estimation.

## Figures and Tables

**Table 1:** Accuracies of different torque classification granularities and preprocessing methods.

Torque Classification Granularity	Dataset	Accuracy for Non-Processed sEMG Signals	Accuracy for Signals Processed by Method A	Accuracy for Signals Processed by Method B
5	Training set Test set	87.18% 86.18%	99% 95.62%	99% 98%
10 15 20	Training set Test set Training set Test set Training set Test se	88.43% 79.37% 89.43% 80.14% 67.37% 30.6%	99% 96.25% 99% 91.25% 99% 66.25%	99% 91.25% 99% 88.12% 99% 64.37%

**Table 2:** Torque classifications and error results.

Classification Granularity	5	11	15	20	25	30	50
Average error	2.488	1.234	0.854	0.861	0.479	0.333	0.249
Maximum error	4.239	2.292	4.399	1.588	2.216	1.428	4.361
Classification granularity	75	100	150	200	350	400	500
Average error	0.174	0.133	0.092	0.070	0.055	0.057	0.049
Maximum error	0.759	2.743	0.945	0.745	6.033	5.900	5.009

**Table 3:** Torque estimation errors before and after applying the MSP-CNN model.

Torque Classification Granularity	Torque Error (N·m)
11 (2D CNN)	1.234
15 (2D CNN)	0.854
Combination of 11 and 15 (MSP-CNN)	0.643
20 (2D CNN)	0.861
25 (2D CNN)	0.479
Combination of 20 and 25 (MSP-CNN)	0.428
30 (2D CNN)	0.333
50 (2D CNN)	0.249
Combination of 30 and 50 (MSP-CNN)	0.216
75 (2D CNN)	0.1739
100 (2D CNN)	0.133
Combination of 75 and 100 (MSP-CNN)	0.105
150 (2D CNN)	0.092
200 (2D CNN)	0.070
Combination of 150 and 200 (MSP-CNN)	0.058
350 (2D CNN)	0.055
400 (2D CNN)	0.057
Combination of 350 and 400 (MSP-CNN)	0.045

**Table 4:** Torque estimation errors by 2D CNN and MSP-CNN using the same classification granularity.

Torque Classification Granularity	Torque Error (N·m)
25 (2D CNN)	0.479
25 (MSP-CNN)	0.243
50 (2D CNN)	0.249
50 (MSP-CNN)	0.137
100 (2D CNN)	0.133
100 (MSP-CNN)	0.070
200 (2D CNN)	0.070
200 (MSP-CNN)	0.043
400 (2D CNN)	0.057
